# Editorial: Virology today in Spain. Selected topics from *Spanish virology*

**DOI:** 10.3389/fcimb.2024.1367322

**Published:** 2024-01-25

**Authors:** Covadonga Alonso, Josep Quer, Isabel García-Dorival

**Affiliations:** ^1^ Departmento de Biotecnología, INIA-CSIC, Centro Nacional Instituto Nacional de Investigación y Tecnología Agraria y Alimentaria, Ctra. de la Coruña, Madrid, Spain; ^2^ Liver Diseases-Viral Hepatitis, Liver Unit, Vall d’Hebron Institut de Recerca (VHIR), Vall d’Hebron Hospital Universitari, Barcelona, Spain; ^3^ Centro de Investigación Biomédica en Red de Enfermedades Hepáticas y Digestivas (CIBERehd), Instituto de Salud Carlos III, Madrid, Spain; ^4^ Biochemistry and Molecular Biology Department, Universitat Autònoma de Barcelona (UAB), Bellaterra, Spain

**Keywords:** virus host, emerging viral infection, hepatitis C virus (HCV), African swine fever virus (ASFV), Adeno associated virus, West Nile virus (WNV), MERS- and SARS-CoV

The field of virology plays a pivotal role in our understanding of “One Health”, encompassing animals, humans and the environment, influencing from public health strategies to advancements in medical treatments. This editorial introduces a series of 10 articles that delve into various aspects of virology, providing readers with a comprehensive and up-to-date overview of the field in this area. It includes interesting original articles about MERS-CoV, West Nile, Usutu, hepatitis C and African swine fever virus, between others, but also includes timely reviews on Coronaviruses (CoV), the origin of pediatric severe acute hepatitis, and antivirals against COVID-19 and human respiratory viruses.

Article by Barrado-Gil et al. investigates the intricate interplay between viruses and their hosts at the molecular level uncovering lipid host factors that influence endocytosis for African swine fever virus infection. Also, an article by García-Crespo et al. explores the mechanisms driving hepatitis C virus (HCV) evolution and adaptation, focusing on the HCV-fitness dependent implications in virus-host interactions and liver disease.

This Research Topic also examines the dynamics of emerging viral threats and discuss the strategies to anticipate and monitor such risks for health.

A clinical threat to the healthcare system was the occurrence of several cases of severe acute hepatitis (not A-E) in 37 countries in July 2022 that the article by Rodríguez-Frias et al. reviews. Their conclusions pointed out that it was caused by Adeno-associated virus 2 (AAV2) infection, associated with a helper virus, and coincident with a determined genetic profile.

The emergence of the largest number of human cases of West Nile virus (WNV) infection occurred in Extremadura, Spain, with six human cases, is reported by Macias et al. These cases occurred in areas were WNV infections have been previously reported, suggesting the definition of an endemic area. Surveillance with early detection methods is encouraged in such regions.

Another research article by Llorente et al., communicates the experimental results regarding the susceptibility of certain bird species, such as red-legged partridges, to WNV strains, while being not competent for transmission of Usutu virus strains.

A review from Luis Enjuanes laboratory, summarizes the current knowledge of human CoV accessory proteins emphasizing their relevant contribution to pathogenesis (Hurtado-Tamayo et al.). Besides, Labiod et al., discuss on the role of DC-SIGN in MERS-CoV dissemination and pathogenesis.

Another study by Fernández-García et al., involved metagenomic sequencing, molecular characterization, and phylogenetics of the vaccine-derived poliovirus strain that caused disease in a patient in Senegal. It was confirmed to be an imported circulating type.

The Research Topic “*Vaccines and Antiviral Therapies*” was also covered by two reports in the antiviral activity of the HRA2pl fusion peptide against paramyxoviruses by Meza et al., and pneumoviruses by and another in the use of Plitidepsin in adult patients of COVID-19 by Varona et al.

This special issue reflects the immense diversity and quality of the research presented and discussed during the congress of the Spanish Society of Virology. Additionally, it serves as a small yet significant example of virology research conducted in Spain, actively promoted by the SEV.

## Author contributions

CA: Conceptualization, Writing – original draft, Writing – review & editing. JQ: Conceptualization, Writing – original draft, Writing – review & editing. IG: Conceptualization, Writing – original draft, Writing – review & editing.

